# Integrated Kinetic Modelling and Microbial Profiling Provide Insights Into Biological Sulfate-Reducing Reactor Design and Operation

**DOI:** 10.3389/fbioe.2022.897094

**Published:** 2022-06-29

**Authors:** Tomas Hessler, Susan T. L. Harrison, Robert J. Huddy

**Affiliations:** ^1^ Department of Chemical Engineering, Centre for Bioprocess Engineering Research (CeBER), University of Cape Town, Cape Town, South Africa; ^2^ Future Water Institute, University of Cape Town, Cape Town, South Africa

**Keywords:** bioremediation, acid rock drainage, microbial ecology, biofilms, sulfate-reducing microorganisms (SRM), 16S rRNA gene amplicon sequencing

## Abstract

Biological sulfate reduction (BSR) is an attractive approach for the bioremediation of sulfate-rich wastewater streams. Many sulfate-reducing microorganisms (SRM), which facilitate this process, have been well-studied in pure culture. However, the role of individual members of microbial communities within BSR bioreactors remains understudied. In this study we investigated the performance of two up-flow anaerobic packed bed reactors (UAPBRs) supplemented primarily with acetate and with lactate, respectively, during a hydraulic retention time (HRT) study set up to remediate sulfate-rich synthetic wastewater over the course of 1,000 + days. Plug-flow hydrodynamics led to a continuum of changing volumetric sulfate reduction rates (VSRRs), available electron donors, degrees of biomass retention and compositions of microbial communities throughout these reactors. Microbial communities throughout the successive zones of the reactors were resolved using 16S rRNA gene amplicon sequencing which allowed the association of features of performance with discrete microorganisms. The acetate UAPBR achieved a maximum VSRR of 23.2 mg.L^−1^. h^−1^ at a one-day HRT and a maximum sulfate conversion of the 1 g/L sulfate of 96% at a four-day HRT. The sulfate reduction reactions in this reactor could be described with a reaction order of 2.9, an important observation for optimisation and future scale-up. The lactate UAPBR achieved a 96% sulfate conversion at one-day HRT, corresponding with a VSRR of 40.1 mg.L^−1^. h^−1^. Lactate was supplied in this reactor at relatively low concentrations necessitating the subsequent use of propionate and acetate, by-products of lactate fermentation with acetate also a by-product of incomplete lactate oxidation, to achieve competitive performance. The consumption of these electron donors could be associated with specific SRM localised within biofilms of discrete zones. The sulfate reduction rates in the lactate UAPBR could be modelled as first-order reactions, indicating effective rates were conferred by these propionate- and acetate-oxidising SRM. Our results demonstrate how acetate, a low-cost substrate, can be used effectively despite low associated SRM growth rates, and that lactate, a more expensive substrate, can be used sparingly to achieve high VSRR and sulfate conversions. We further identified the preferred environment of additional microorganisms to inform how these microorganisms could be enriched or diminished in BSR reactors.

## Introduction

Sulfate contaminated wastewater streams are generated by numerous industries including coal power generation (Johnson and Santos, 2000), several chemical industries (Sarti and Zaiat, 2011) and mining in the form of acid mine drainage (AMD; Johnson and Hallberg, 2005). Biological sulfate reduction (BSR) has been shown to be a sustainable bioremediation option for the treatment of sulfate-rich wastewater streams (Lens et al., 2002). This is a particularly appealing for the remediation of AMD due to its low cost and ability to be operated alongside the generation of AMD in perpetuum. BSR can simultaneously valorise AMD, with reactors developed for the recovery of metals (Hedrich and Johnson, 2014) and elemental sulphur (Marais et al., 2020).

BSR relies upon sulfate-reducing microorganisms (SRM; Muyzer and Stams, 2008), a diverse group of anaerobic microorganisms represented across at least 22 phyla (Anantharaman et al., 2018), to reduce sulfate present in the wastewater to sulfide through the process of dissimilatory sulfate reduction. This sulfate reduction is coupled to the oxidation of supplied electron donor, typically volatile fatty acids (VFAs) such as lactate, ethanol, acetate and H_2_ (Liamleam and Annachhatre, 2007). The supplemented electron donor makes up a large proportion of the associated operating costs (Papirio et al., 2013). Careful selection of electron donors and operating conditions, therefore, is essential to ensure adequate sulfate reduction rates and conversions are achieved with efficient electron donor utilisation.

Acetate is an appealing electron donor for BSR (Eq 1; Table 1) due to its low cost (0.6 USD/kg; LePro Pharma Compass, n. d.,a) and it being present in a variety of waste streams (Liamleam and Annachhatre, 2007). However, the low supported microbial growth rates using acetate (Thauer et al., 1977) can make achieving suitably high reaction rates difficult. This has previously been overcome by decoupling the biomass- and the hydraulic retention time (HRT), allowing accumulation of biomass within BSR reactors (Harada et al., 1994).

**TABLE 1:** Common sulfate-reducing and lactate fermenting reactions, adapted from Thauer et al. (1977).

Reaction Equation	ΔG°’ (kJ/Reaction)	
Acetate^−^ + SO_4_^2−^ → 2 HCO_3_^−^ + HS^−^	−47.6	Eq (1)
Propionate^−^ + 0.75 SO_4_^2−^ → Acetate^−^ + HCO_3_^−^ + 0.75 HS^−^ + 0.25 H^+^	−37.7	Eq (2)
Lactate^−^ + 0.5 SO_4_^2−^ → Acetate^−^+ HCO_3_^−^ + 0.5 HS^−^	−80.2	Eq (3)
2 Lactate^−^ + 3 SO_4_^2−^ → 6 HCO_3_^−^ + 3 HS^−^ + H^+^	−225.3	Eq (4)
3 Lactate^−^ → Acetate^−^ + 2 Propionate^−^ + HCO_3_^−^ + H^+^	−70.0	Eq (5)

Lactate is a well-established electron donor for BSR as it provides high sulfate reduction rates and can be used by a wide range of SRM which oxidise it to acetate (Eq 3) or completely to CO_2_ (Eq 4). Unfortunately, it is expensive (2.4 USD/kg; LePro Pharma Compass, n. d.,b) and is consumed readily by fermentative microorganisms (Eq 5) which can make it unappealing for industrial applications. Kinetic investigations have found SRM can outcompete the fermentative microorganisms, with greater characterised μ_max_, at low substrate concentrations due to higher characterised affinities of SRM for lactate (Oyekola et al., 2009, 2012).

The physiology of dissimilatory sulfate reduction (Santos et al., 2015), the identification of SRM able to grow under stressed conditions and the expanded applications of BSR (Qian et al., 2019) have seen continual progress. However, the ecology of the communities in BSR reactors and their relation to performance remains understudied and a consensus on the desired community has not sufficiently been reached. Phenotypic characterisations of SRM in pure culture have provided a detailed understanding of the electron donors available to different SRM, however, confirmation that these SRM can effectively compete for these electron donors within the competitive mixed microbial reactor environment is seldom addressed.

In this study, we operated two continuous up-flow anaerobic packed-bed reactors (UAPBRs) during an HRT study using a synthetic waste stream containing 1.0 g/L sulfate and supplemented with acetate and with lactate, respectively, as the primary electron donors. The nature of the sulfate reduction reactions were investigated by modelling the observed sulfate reduction as irreversible reactions within ideal plug flow reactors and by associating the observed sulfate reduction with the observed oxidation of several electron donors. The contribution of the microbial communities towards the observed reactions were investigated by evaluating their discrete biomass concentrations throughout the successive zones of the reactors and by assessing the microbial composition through 16S rRNA gene amplicon sequencing. This was performed to attempt to link physiochemical observations such as sulfate reduction and electron donor utilisation occurring in specific zones to particular identified microorganisms.

We previously made such attempts based on the performance and supported microbial communites associated with this lactate UAPBR at a four-day HRT (Hessler et al., 2018). The current study continues these observations whilst the reactor is subjected to a HRT study and is compared with an identical reactor operated with acetate as the primary electron donor.

In addition to the two UAPBRs which are described in the current study, we simultaneously inoculated and operated two continuous stirred-tank reactors and two linear flow channel reactors. Some of these data have been described previously (Hessler et al., 2020). The predominant operational taxonomic units (OTUs) identified in the two described UAPBRs were further investigated by assessing their distribution in characterizable environments across all six BSR reactors in order to discern the conditions in which these microorganisms were most prevalent.

Lastly, in a previous work we performed genome-resolved metagenomics on samples from these six bioreactors at a four-day HRT (Hessler et al., 2021). In this metagenomic study we recovered and assembled 163 recovered microbial genome bins and presented analysis of their encoded metabolisms. We make reference to this work during the current study to substantiate links we draw between microorganisms we identified within this study using 16S rRNA amplicon sequencing and their possible roles in these UAPBRs.

## Methodology

### Microbial Culture and Inoculation

The reactors were inoculated with a sulfidogenic mixed microbial culture originally obtained from the Microbiology, Biochemistry and Biotechnology Department at Rhodes University in 2001 and subsequently complemented with biological material from an industrial oxidation pond and anoxic river sediment. This culture has since maintained as batch cultures and separately supplemented with different electron donors. These batch cultures were combined and inoculated at a 1:1 ratio with neutral Postgate B media (0.42 g/L KH_2_PO_4_, 1.0 g/L NH_4_Cl, 1.0 g/L MgSO_4_.7H_2_O, 0.9 g/L Na_2_SO_4_, 0.4 g/L yeast extract, 0.3 g/L sodium citrate) containing with 1.0 g/L (10.4 mM) sulfate supplemented with either 0.92 g/L sodium acetate (15.3 mM) or 1.2 g/L sodium lactate (13.6 mM) as the primary electron donors. Citrate and yeast extract in the media too are known electron donors for SRM but were incorporated to prevent media precipitation and as a source of amino acids and cofactors, respectively. Sodium 2-bromoethanesulfonate (BESA), a methanogenic inhibitor, was added to each reactor at a final concentration of 10.0 mM at the time of inoculation.

### Reactor Systems and Operation

**FIGURE 1:**
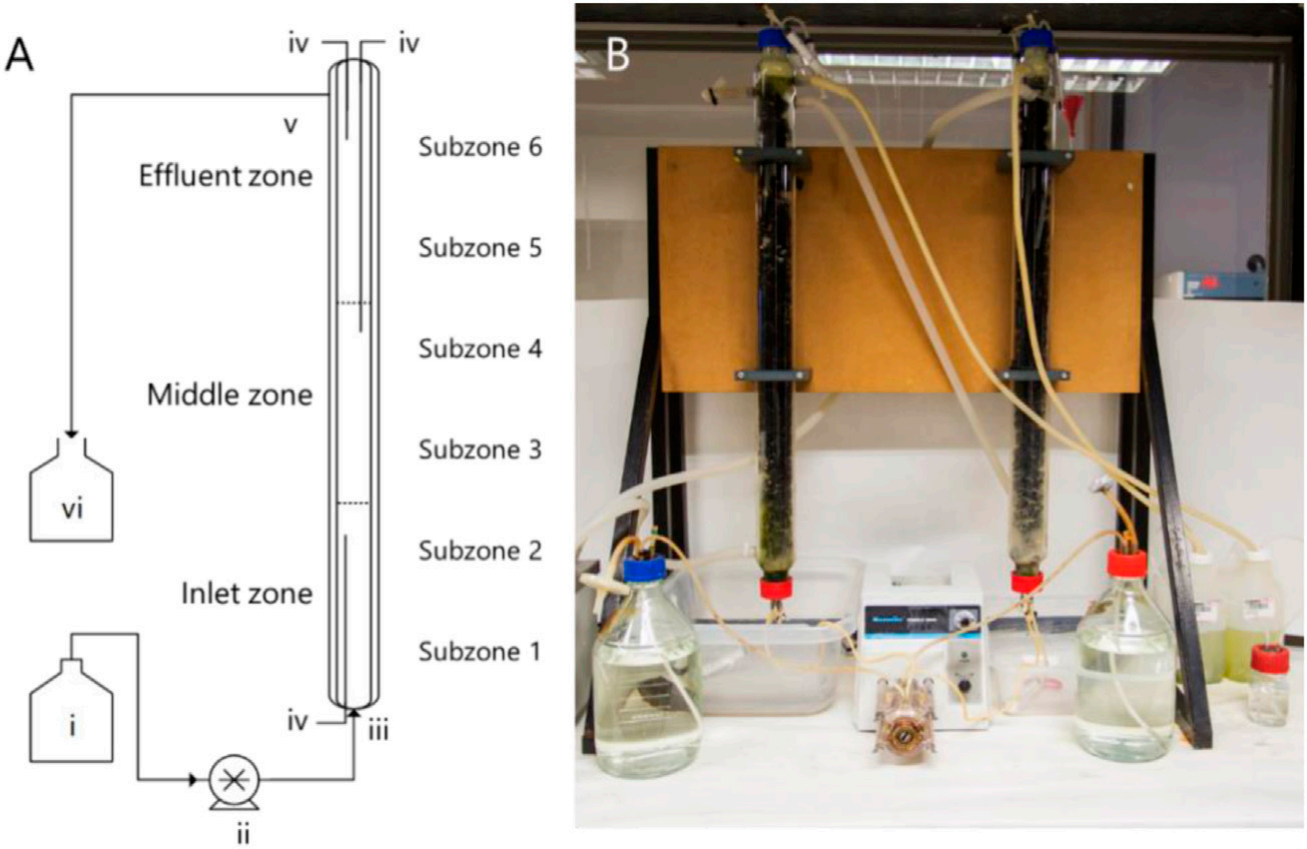
**(A)** Schematic diagram and **(B)** photograph of the UAPBRs operated in this study, labelled with the layout of the demarcated zones and the (i) feed reservoir, (ii) peristaltic pump, (iii) inlet port and (iv) sampling ports. The reactors were discharged by gravity via the (v) effluent port and waste collected in (vi).

The UAPBRs used in this study are described in Hessler et al. (2018). Briefly, these UAPBRs had a working volume of 1.0 L and were packed with open-pore polyurethane foam pieces which displaced a total of 40 ml (4%) of the working volume of the reactors. The polyurethane foam was cut to 2.0 cm^3^ from polyurethane sheets manufactured as conventional fish tank filters for indoor aquaria. The reactors were demarcated into three sequential zones of 0.33 L, referred to as the inlet-, middle- and effluent-zones (Figure 1). Sampling ports positioned at the bottom and top of the reactor extended to the boundary between the between each zone and just below the effluent port. These zones were sampled independently for solution chemistry and planktonic cells throughout the experimental period. The reactors internal temperature were maintained at 30 °C through the use of glass jackets and circulating water baths. The reactors were further demarcated into six sequential subzones (V = 0.167 ml) for greater resolution when assessing the biofilm communities throughout the height of these reactors. Following inoculation, the headspace of the UAPBRs were sparged with nitrogen gas and were then operated as batch systems for one week before supplying the corresponding Postgate B media to the reactors via in the inlet ports at an initial four-day HRT (0.010 h^−1^). The reactors were initially operated with 1.0 g/L yeast extract but this was reduced to 0.4 g/L on day 346. Upon steady-state conditions, defined as consistent sulfate reduction over a period of at least three HRTs, the applied dilution rate was iteratively increased to 0.014, 0.016, 0.018, 0.021, 0.024, 0.028, 0.032 h^−1^ and a final dilution rate of 0.042 h^−1^ (one-day HRT). A minimum of five samples were taken for solution chemistry during each defined steady-state period. These reactors were operated for a total of 1,032 days between inoculation and steady-state at a one-day HRT.

### Analytical Methods

The produced sulfide and residual sulfate were assayed using the N,N-dimethyl-p-phenylenediamine (Cline, 1969) and APHA turbimetric (Greenberg and Eaton, 1999) methods, respectively. The residual VFAs, including citrate, lactate, butyrate, valerate, propionate and acetate were quantified using a Waters Breeze 2 high-performance liquid chromatography (HPLC) as described by Hessler et al. (2018). Redox potential and pH measurements of each sample were determined using a Cyberscan 2,500 micro pH meter fitted with an XS Sensor 2-Pore T DHS pH 130 probe, and a Metrohm 827 pH lab meter 131 fitted with a Pt-ring KCl electrode (Metrohm model 6.0451.100), respectively (Supplementary Table S2 and Supplementary Table S5). A minimum of four temporarily separated samples, for each analytical measurement, were taken during each defined steady state period.

### Steady-State Biological Sampling

At each steady state, cells from each of the three defined microbial phases, namely planktonic as well as matrix-attached and matrix-associated phases, were recovered separately for biomass quantification and total genomic DNA extraction. Planktonic cells were recovered via sampling ports, in duplicate, from each 0.33 L reactor zone at steady-state at each tested dilution rate tested. Cells were quantified by direct microscope cell counting, performed in duplicate, as described by Hessler et al. (2018). Two representative pieces of colonised foam were used for total genomic DNA extraction. The biofilm cells firmly attached (“biofilm-attached”) and those weakly attached (“biofilm-associated”) to the polyurethane supports were separately recovered for biomass quantification using a non-destructive whole-cell detachment protocol (Hessler et al., 2018). All biofilm cell concentrations were calculated relative to the volume of a reactor subzone (0.167 L) minus the volume displaced by the polyurethane foam (4%). Briefly, three pieces of polyurethane foam were independently isolated, in duplicate and at random, from each of the six sequential reactor subzones. The sampling of the colonised foam was repeated, as described above, for the isolation of total genomic DNA. The biofilm-associated communities were isolated from colonised foam pieces using the detachment protocol and recovered by centrifugation at 10,000 g for 10 min. Total genomic DNA from the attached cells was then extracted directly off this polyurethane foam.

### Modelling of UAPBR Kinetic Data

Sulfate reduction rates were initially calculated using Eq. 6. Sulfate-reducing reaction rate data collected from each UAPBR over the course of the study were modelled as irreversible n^th^-order and first-order reactions (Eq 6) along an ideal plug flow reactor (Eq 7) according to derived Eq. 8 and Eq. 9, respectively, where *rA* is the sulfate reduction reaction rate (mg.L^−1^. h^−1^), *V* is the volume (L) of the reactor or zone, *X* is the observed sulfate conversion, *F* is the applied flow rate (L.h^−1^) which is equal to reactor volume divided by the HRT, *C*_*0*_ is the concentration of sulfate entering the reactor or zone (mg/L), *C*_*A*_ is the observed concentration of sulfate leaving the reactor or zone (mg/L), *n* is the reaction order and *k* is the rate constant. The derivation of Eqs 8, 9 are documented in the Supplementary Methods.

$$\frac{dX}{dV}=\frac{-rA}{F.C_{0}}$$ (6)

$$rA=-k.C_{A}^{n}$$ (7)

$$rA=\frac{V}{F}\;\left({\left(C_{0}^{\left(-n+1\right)}+\left(n-1\right)\cdot k\cdot \frac{V}{F}\right)}^{\frac{1}{\left(-n+1\right)}}-C_{0}\right)$$ (8)

where n ≠ 1 Eq. 8

$$rA=-\frac{F}{V}\left(\frac{C_{0}}{\frac{V.k}{{\text{e}}^{\text{F}}}}-C_{0}\right)$$ (9)

Non-linear regression using the iterative, generalised reduced gradient (GRG) method, employed by SOLVER (Microsoft Excel, Microsoft Office 365 ProPlus) was used to solve for the rate constant and reaction order (Warren Lee and Smith, 1976). This method varies *k* and *n* to yield the lowest possible standard squared error (SSE; Eq 10) against the observed reaction rate, where *y* is the observed reaction rate and *y*_*fit*_ is the modelled reaction rate. The goodness of fit of each modelled dataset was performed by calculating the coefficient of determination *R* ^*2*^ (Kvalseth, 1983) as shown in Eq. 11, where *y*_*mean*_ is the average of all observed reaction rates (*rA*). Confidence intervals were determined by multiplying the standard error (Eq 12) by the T-critical value determined using a two-tailed inverse of the Student’s t-distribution using a 95% probability.

$$SSE=\sum {\left(y-y_{fit}\right)}^{2}$$ (10)

$$R^{2}=1-\frac{\sum {\left(y-y_{fit}\right)}^{2}}{\sum {\left(y-y_{mean}\right)}^{2}}$$ (11)

$$SE=\sqrt{\frac{SSE}{\deg rees\;of\;freedom}}$$ (12)

### DNA Extraction and 16S rRNA Gene Amplicon Sequencing

Total genomic DNA was extracted from the planktonic, biofilm-associated and -attached communities from the successive zones of the two UAPBRs using a NucleoSpin^®^ soil genomic DNA extraction kit (Machery-Nagel, Germany) as per manufacturer’s instructions. The V3 - V4 region of the 16S rRNA gene was amplified from total genomic DNA by Polymerase Chain Reaction using primers and conditions as described by Hessler et al. (2018). The generated amplicon libraries were sequenced on an Illumina^®^ MiSeq^®^ to yield 300 bp paired-end reads.

### Microbial Community Analysis

Illumina^®^ read processing, the picking of operational taxonomic units (OTU) and taxonomic classification of the OTUs was performed as described by Hessler et al. (2018). The 16S rRNA gene sequences have been deposited at GenBank under the accession numbers: MH603613-MH603682. Similarly the raw reads can be found under the Bioproject accession number PRJNA836584. Total genomic DNA extracted from the communities within subzone six of both UAPBRs, a one-day HRT, were sequenced in duplicate. Variation in OTU abundances between these repeats were evaluated by calculating coefficients of variation (Supplementary Table S7) and presented as rank abundance curves (Supplementary Figure S4). The depth of sequencing and the alpha diversity of each microbial community was estimated using goods coverage and Chao1 richness estimator, Shannon indices and Simpson indices, respectively Supplementary Table S8). Hierarchical clustering of OTU abundances across UAPBR microbial communities was performed using Ward’s method and Euclidean distances by Clustvis (Metsalu and Vilo, 2015). The beta-diversity between communities were estimated by calculating weighted-Unifrac distances (Lozupone and Knight, 2005) using QIIME (Caporaso et al., 2010) and assessed using principal component analysis using Clustvis. The relative abundance of each OTUs across all UAPBR samples can be found in the supplementary material (Supplementary Table S10).

### Enrichment of OTUs Across Physiochemical Environments

The enrichment of select OTUs, when present (0.01% relative abundance cut-off) in a community, under mutually exclusive sets of physicochemical conditions was tested using Walds t-tests assuming unequal variance. The tested environments were biofilm-attached versus planktonic communities, and zones where lactate was available versus absent. The gene survey encompassed the UAPBRs’ communities, as well as those of duplicate acetate and lactate supplemented continuously stirred tank reactors (CSTRs) (Hessler et al., 2018) and Linear flow channel reactors (LFCRs) (Hessler et al., 2020), inoculated simultaneously and operated under identical conditions.

## Results

### Sulfate-Reducing Performance

**FIGURE 2:**
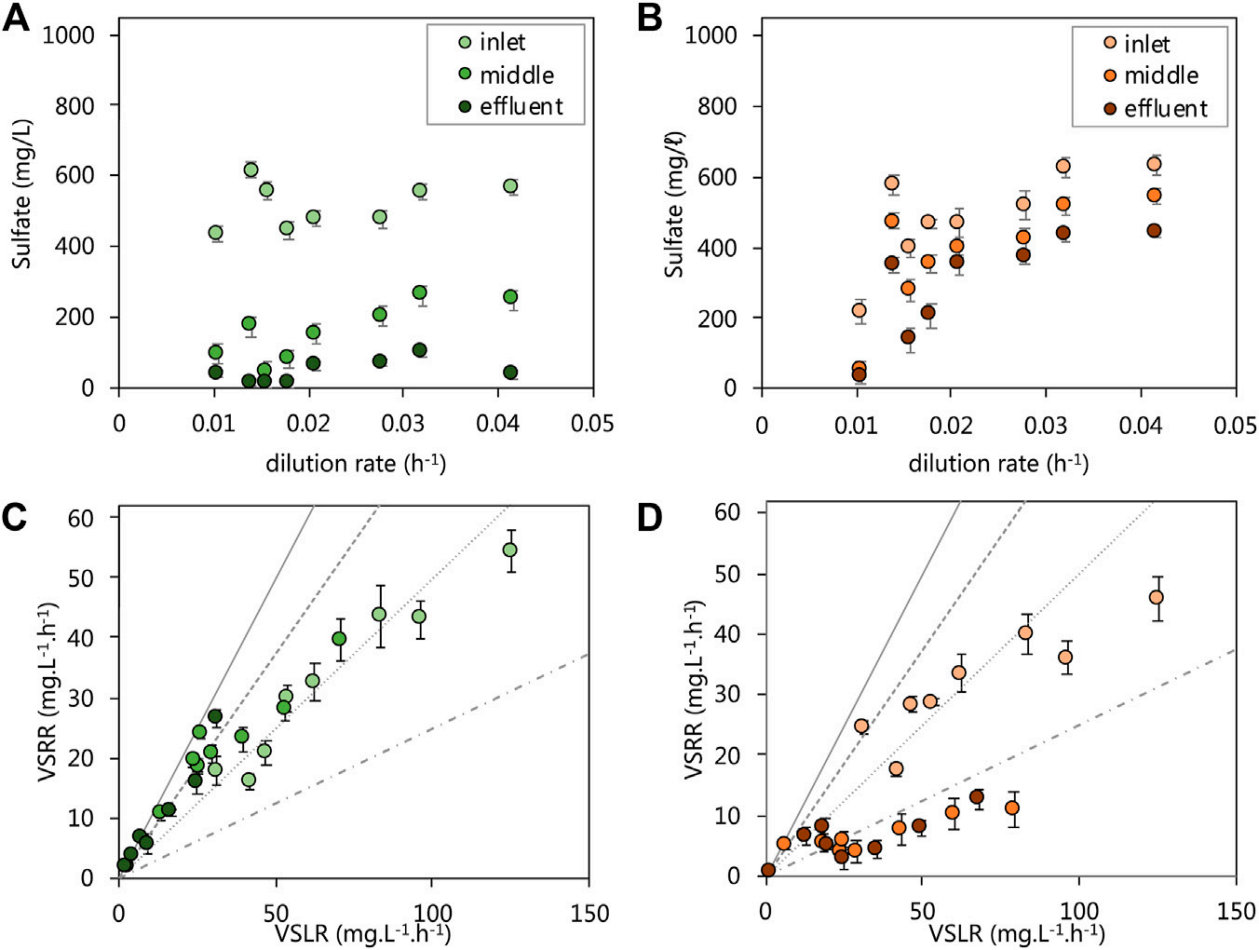
Steady-state residual sulfate concentration leaving each zone of the **(A)** lactate and **(B)** acetate reactors and the VSRRs achieved by each zone of the **(C)** lactate and **(D)** acetate reactors against the applied VSLRs. Sulfate conversion can be visually be determined through comparisons with lines plotted: 100% conversion–solid line, 75% conversion–dashed line; 50% conversion–dotted line; 25% conversion–composite dotted-dashed line. Error bars represent one standard deviation from the mean (*n* > 4).

We evaluated the performance of the acetate- and lactate-supplemented UAPBRS primarily by their ability to remove sulfate from solution over the course of the HRT study. The lactate-supplemented UAPBR maintained a sulfate conversion of >90.0% for the duration of the study and achieved a 96.4% conversion at the final HRT of one-day (0.042 h^−1^ dilution rate, Figure 2A), corresponding to a maximum observed VSRR of 40.1 mg.L^−1^. h^−1^. Between 550 and 600 mg/L (5.73–6.25 mM) of 1,000 mg/L sulfate was consumed within the inlet zone throughout the study (Figure 2A), excluding 0.014–0.016 h^−1^ tested dilution rates where consumption decreased to 350 and 400 mg/L (3.65 and 4.17 mM) sulfate, respectively. The sulphate conversion then improved following this period of temporary poor performance. We, therefore, excluded these data points from the sulfate reduction rate modelling as this indicated that a true steady state was not achieved following the perturbation brought on by the increase of the dilution rate. The highest VSRR exhibited by the inlet zone was 53.4 mg.L^−1^. h^−1^, at a 0.042 h^−1^ dilution rate (one-day HRT; Figure 2C). The middle zone of the lactate- UAPBR consistently reduced between 277 and 511 mg/L (2.89–5.32 mM) of sulfate (Figure 2A) and was able to achieve sulfate conversions of 53–92% of the sulfate which entered zone (Figure 2C). Little sulfate (<40 mg/L) entered the effluent zone of the reactor until a dilution rate of 0.021 h^−1^ was reached. The majority of the sulfate which entered the effluent zone was then consumed from this time-point (Figures 2A,C).

The acetate UAPBR initially showed very high sulfate conversions, but unlike the lactate UAPBR, this was not maintained at reduced HRT. This reactor saw a sulfate conversion of 96% over the length of the reactor at a four-day HRT (0.010 h^−1^ dilution rate) with 75% of this sulfate consumed within the inlet zone (Figure 2B). The acetate UAPBR inlet zone did not respond well to the first increase in applied dilution rate. The sulfate conversion exhibited by this zone subsequently improved from 38.5% at a 0.014 h^−1^ dilution rate to 60% at a 0.016 h^−1^ dilution rate. Likewise, these data were excluded from kinetic modelling as we believe these not to be true steady states. The inlet zone of the acetate UAPBR performed similarly to that of the lactate UAPBR, despite sulphate reduction coupled to lactate oxidation being a far more favourable than that linked to acetate oxidation (Table 1). The VSRR exhibited by the inlet zone reached a maximum of 45.8 mg.L^−1^. h^−1^ at a 0.042 h^−1^ dilution rate (one-day HRT). The overall sulfate conversion of the acetate reactor decreased to approximately 60% at a 0.032 h^−1^ dilution rate and remained constant at a 0.042 h^−1^ dilution rate. The highest VSRR exhibited over the length of this reactor was 23.2 mg.L^−1^. h^−1^ observed at a 0.042 h^−1^ dilution rate. The middle and effluent zones of the reactor, together, over the course of the study, removed 150–250 mg/L (1.56–2.60 mM) of sulfate, far less than removed in the inlet zone.

### Electron Donor Utilisation in the Lactate-Supplemented UAPBR

**FIGURE 3:**
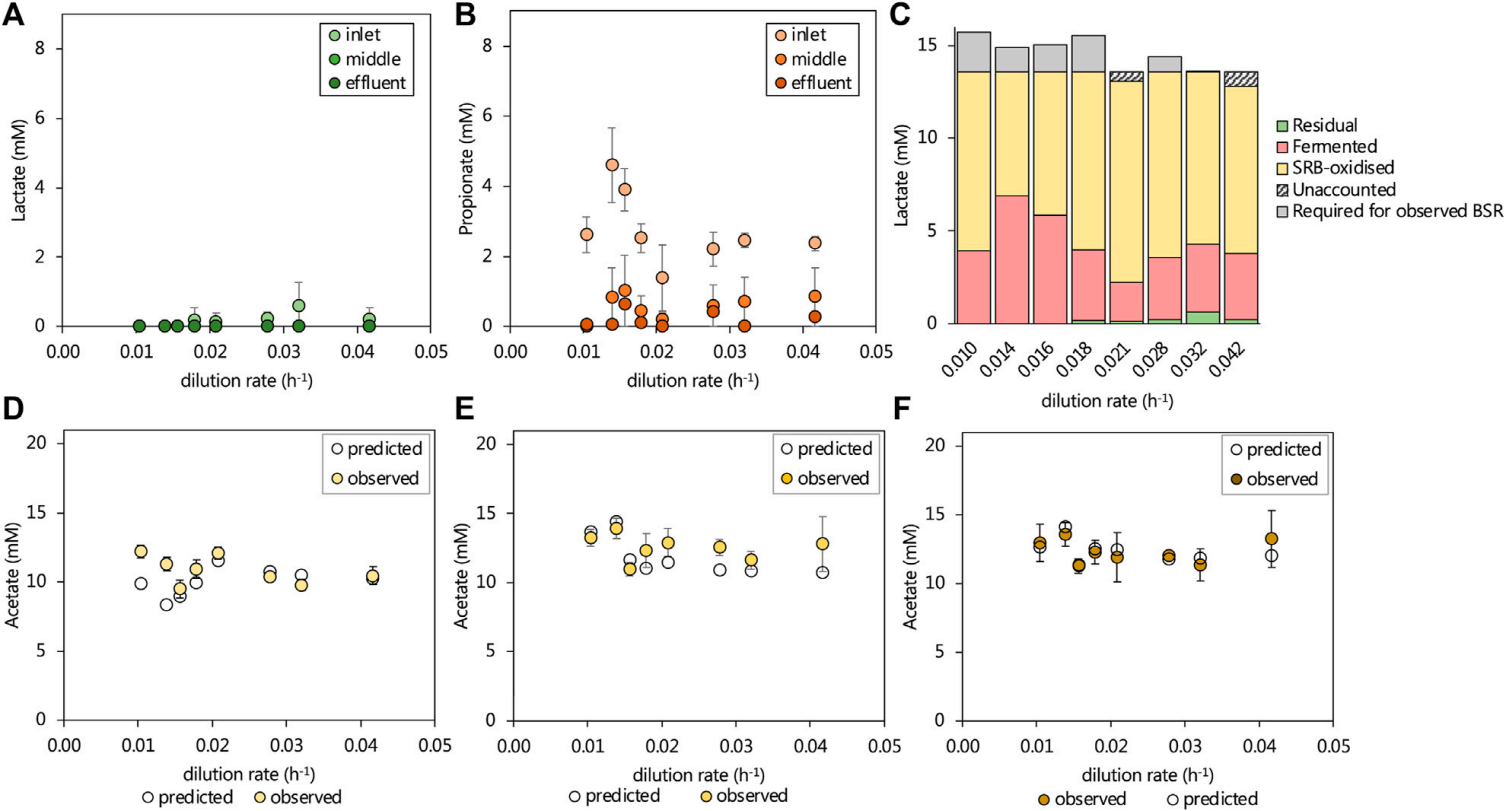
The observed steady-state residual **(A)** lactate and **(B)** propionate leaving the three zones of the lactate UAPBR at increasing dilution rates. The **(C)** amount of lactate fermented (Eq. 5) was estimated based on the residual propionate concentration and the remainder of available lactate was assumed to be consumed by SRM and verified based on the degree of sulfate reduction. Where more sulfate reduction occurred than could be stoichiometrically accounted for by incomplete lactate oxidation by SRM, this was indicated as lactate “required for observed BSR”. The observed and predicted acetate concentrations leaving the **(D)** inlet **(E)** middle and **(F)** effluent zones of the lactate-supplemented UAPBR are shown. The predicted acetate concentrations were calculated based on the assumptions described in text and that the oxidation of 0.4 g/L yeast extract led to the generation of 268 mg/L acetate, as described in Hessler et al. (2021). Error bars represent one standard deviation from the mean (*n* > 4).

The 13.3 mM lactate supplied to the lactate UAPBR theoretically allowed for only 64% of the 1.0 g/L (10.42 mM) sulfate to be reduced through incomplete lactate oxidation by SRM (Eq. 3). Further sulfate reduction required the oxidation of produced propionate and acetate according to Eqs. 1, 2, respectively. Over 95% of lactate supplied to the UAPBR was consumed within the inlet zone at each tested dilution rate (Figure 3A) as well as all of the 1.16 mM citrate. Propionate was consistently detected leaving the inlet zone, typically at approximately 2.3 mM. At dilution rates of 0.014 and 0.016 h^−1^, this propionate concentration had increased to 4.6 and 3.9 mM, respectively, and corresponded with the reduced sulfate-reducing performance of the inlet zone at these steady-states. Little to no propionate was detected in the acetate-supplemented UAPBR and, therefore, all observed propionate in the lactate-UAPBR was linked to the fermentation of the supplied lactate according to Eq. 5 (Figure 3C). This suggested that approximately 25% of the supplied lactate was utilised by fermentative microorganisms at the initial tested HRT. However, at dilution rates of 0.014 and 0.016 h^−1^, this had increased to 51 and 43% respectively.

The degree of sulfate reduction which occurred in the inlet zone at each tested dilution rate was linked to the oxidation of the remaining available lactate according to Eq. 3. Where more sulfate reduction had occurred than could be accounted for by the observed lactate oxidation, the subsequent oxidation of produced acetate was assumed according to Eq 1 and 4 (Figure 3C).

The majority of the propionate produced in the inlet zone was subsequently consumed within the middle zone of this reactor at each tested dilution rate. The sulfate reduction which occurred within the middle zone could, therefore, be linked primarily to the oxidation of propionate and secondly to the oxidation of acetate.

The oxidation of the 0.4 g/L yeast extract, supplied in the medium as a source of micronutrients, was previously found to consistently produce 268 mg/L acetate (Hessler et al., 2021). This estimation could be made after the yeast extract concentration was reduced from 1.0 to 0.4 g/L. This reduction in yeast extract was also found to have minimal impact on the performance of these reactors and led us to conclude that yeast extract was not the primary electron donor for sulfate reduction and was more likely consumed by the present fermentative microorganisms and producing acetate.

Informed by this, the expected acetate concentrations were calculated based on the contribution of the deduced lactate and propionate oxidation and lactate fermentation reactions together with an additional 268 mg/L acetate originating from yeast extract, with any remaining unaccounted for sulfate reduction assumed to be linked to acetate oxidation (Eq 1). These predictions are plotted against the observed acetate concentration in each zone, at each tested dilution rate in Figures 3D–F. Within the inlet, middle and effluent zones the observed and predicted acetate concentrations differed, on average across dilution rates, by just 6.9, 11.6 and 0.3% of the observed acetate concentration.

### Electron Donor Utilisation in the Acetate-Supplemented UAPBR

**FIGURE 4:**
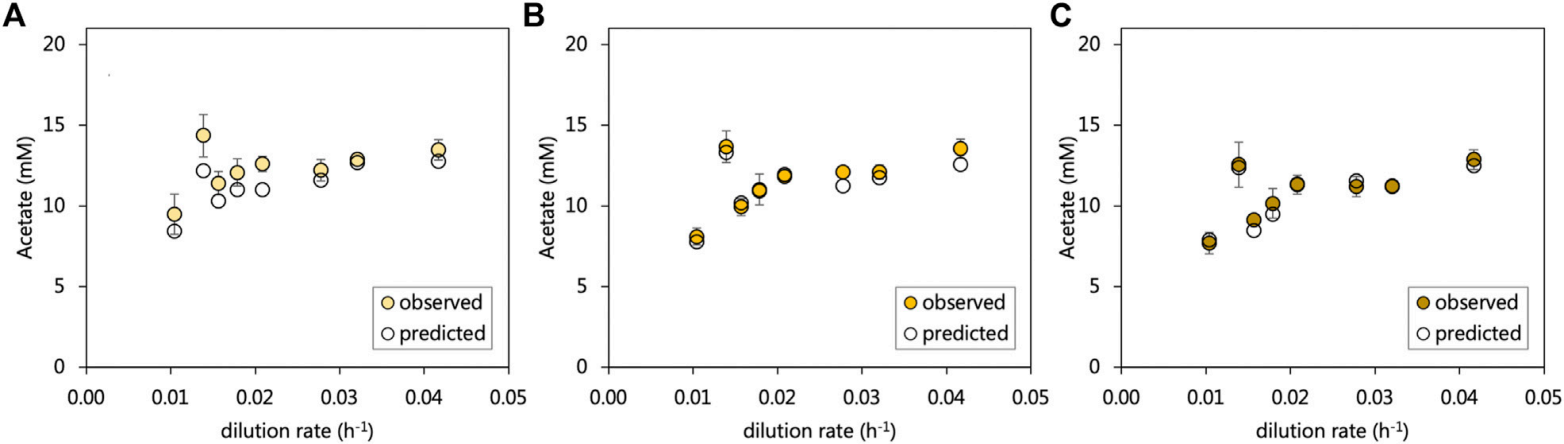
The observed and predicted acetate concentrations leaving the **(A)** inlet **(B)** middle and **(C)** effluent zone of the acetate-supplemented UAPBR. Acetate concentrations were predicted with the assumptions that (i) all sulfate reduction observed was linked to the oxidation of acetate according to Eq. 1, and (ii) that the oxidation of 0.4 g/L of yeast extract led to the production of 268 mg/L of acetate (Hessler et al., 2020). Error bars represent one standard deviation from the mean (*n* > 4).

The residual acetate concentration leaving the inlet zone decreased by on average, 0.8 mM in the middle zone and subsequently 0.8 mM in the effluent zone. An overall increasing trend in residual acetate was evident with the increasing dilution rate, which corresponded with decreasing sulfate conversions. The particularly poor sulfate-reducing performance of the inlet zone at a 0.014 h^−1^ dilution rate corresponded with a sudden increase in the residual acetate concentration. All sulfate reduced in the acetate-UAPBR was assumed to be linked to the oxidation of acetate according to Eq. 1. Expected acetate concentrations were calculated for each zone and including the generation of 268 mg/L acetate in the inlet zone through yeast extract fermentation. Within the inlet, middle and effluent zones the observed and predicted acetate concentrations differed, on average across tested dilution rates, by 8.7, 7.3, and 2.1% of the observed acetate concentration (Figure 4), suggesting that sulfate reduction is coupled primarily to acetate oxidation. The additional, unaccounted-for acetate which almost exclusively observed in the inlet zone, could also have resulted from the oxidation of 1.16 mM citrate by non-sulfate-reducers, as its oxidation was only observed within this zone, and was not detected within the reactor at any HRT.

### Sulfate-Reducing Models

**FIGURE 5:**
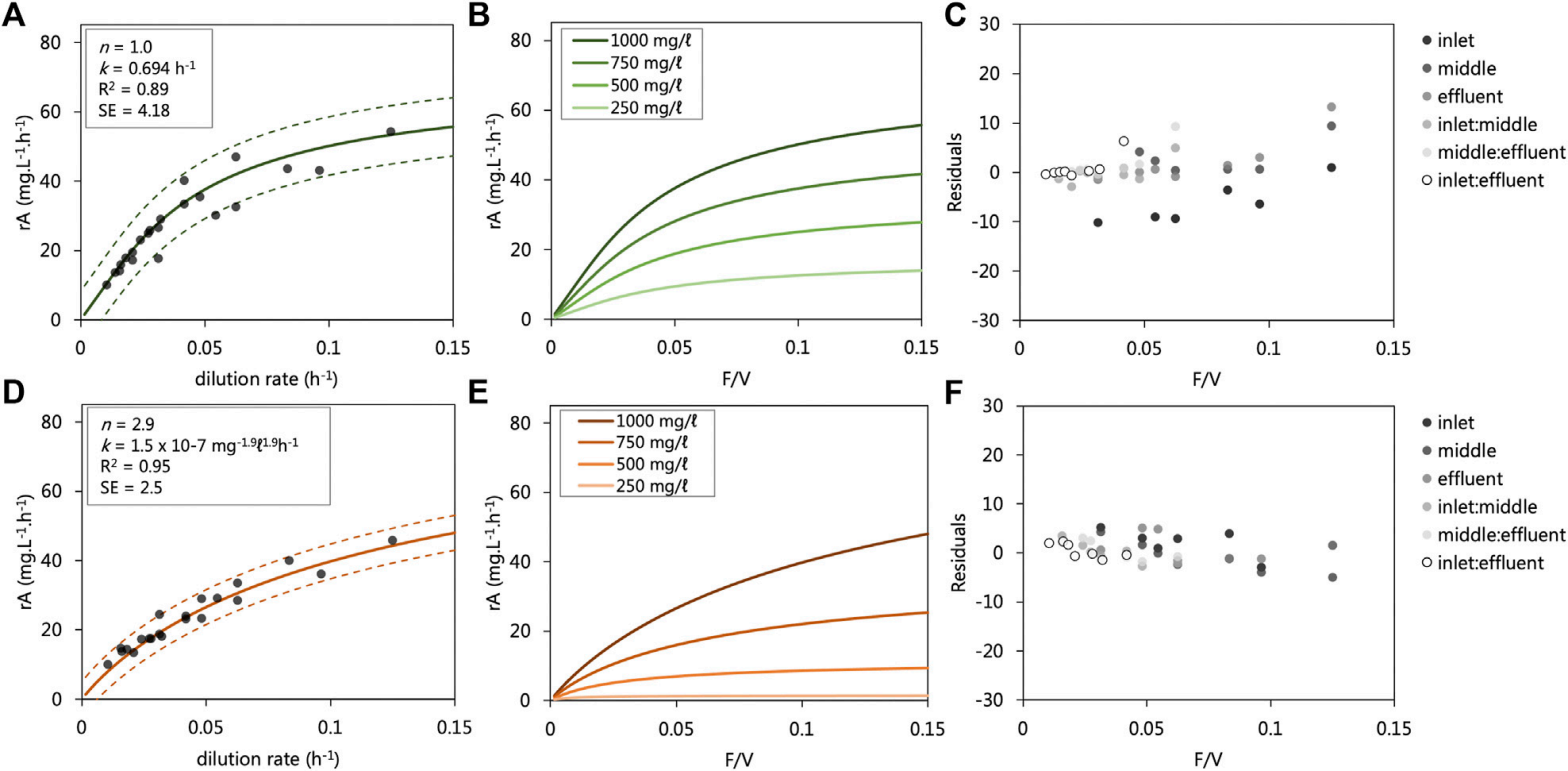
Modelled sulfate reduction rates observed in the **(A,B)** lactate- and **(D,E)** acetate-supplemented UAPBRs at a range of flow rate to volume ratios (F/V or dilution rates). The modelled reaction rates are shown over a range of applied dilution rates where the starting substrate concentration (C_0_) is 1,000 mg/L **(A,B)** with observed reaction rate data from the inlet zones (0.33 L), composite inlet and middle zones (0.66 L) and entire reactors (1.0 L). The determined reaction order (*n*), rate constant (*k*), *R* ^2^ value and standard error (SE) calculated for each of the modelled datasets are shown. The residuals of the modelled minus observed sulfate reduction rates are shown for the **(C)** lactate and **(F)** Acetate UAPBRs. The relation between the observed and modelled reaction rates of the entire dataset is shown in Supplementary Figures S2,S3. C; **(D)** Modelled reaction rate data at various flow rate to volume ratios (F/V) with various starting sulfate starting concentrations (C_*0*_) is shown. 95% confidence intervals are shown as dashed lines.

The kinetic data collected from each UAPBRs were modelled as irreversible reactions in ideal plug-flow reactors according to Eq 8 and 9. The relation between the observed and modelled reaction rates of the entire dataset, together with their residual, are shown in Figures 5C,F. The modelled reaction rate data collected from the lactate-UAPBR found these reactions could be described as a first-order reaction with rate constant equal to 0.06955 h^−1^ (Figures 5A,B). The largest residuals (difference between observed and predicted) were observed for the reaction rates of the inlet zone at short dilution rates, where the model overpredicted the VSRR occurring in this zone. We attribute this to a volume of sludge which had collected at the base of the reactor (Figure 5C) causing a reduction in the volume of the zone. The first-order nature of the overall reaction indicates the magnitude of the reaction is proportional only to the concentration of sulfate. The oxidation of lactate, restricted to the inlet zone, is more favourable than the oxidation of propionate and acetate, occurring mainly in the middle and effluent zones, according to Eq’s (1–3).

The sulfate-reduction reactions observed in the acetate-supplemented UAPBR could be described with a reaction order of 2.9 and a rate constant of 1.5 × 10^−7^ mg^−1.9^L^1.9^h^−1^ (Figures 5D,E). The high reaction order indicates that although sulfate may have been consumed readily at high concentrations, the VSRR decreases exponentially with decreasing sulfate concentrations (Figure 5D).

### Biomass Retention

**FIGURE 6:**
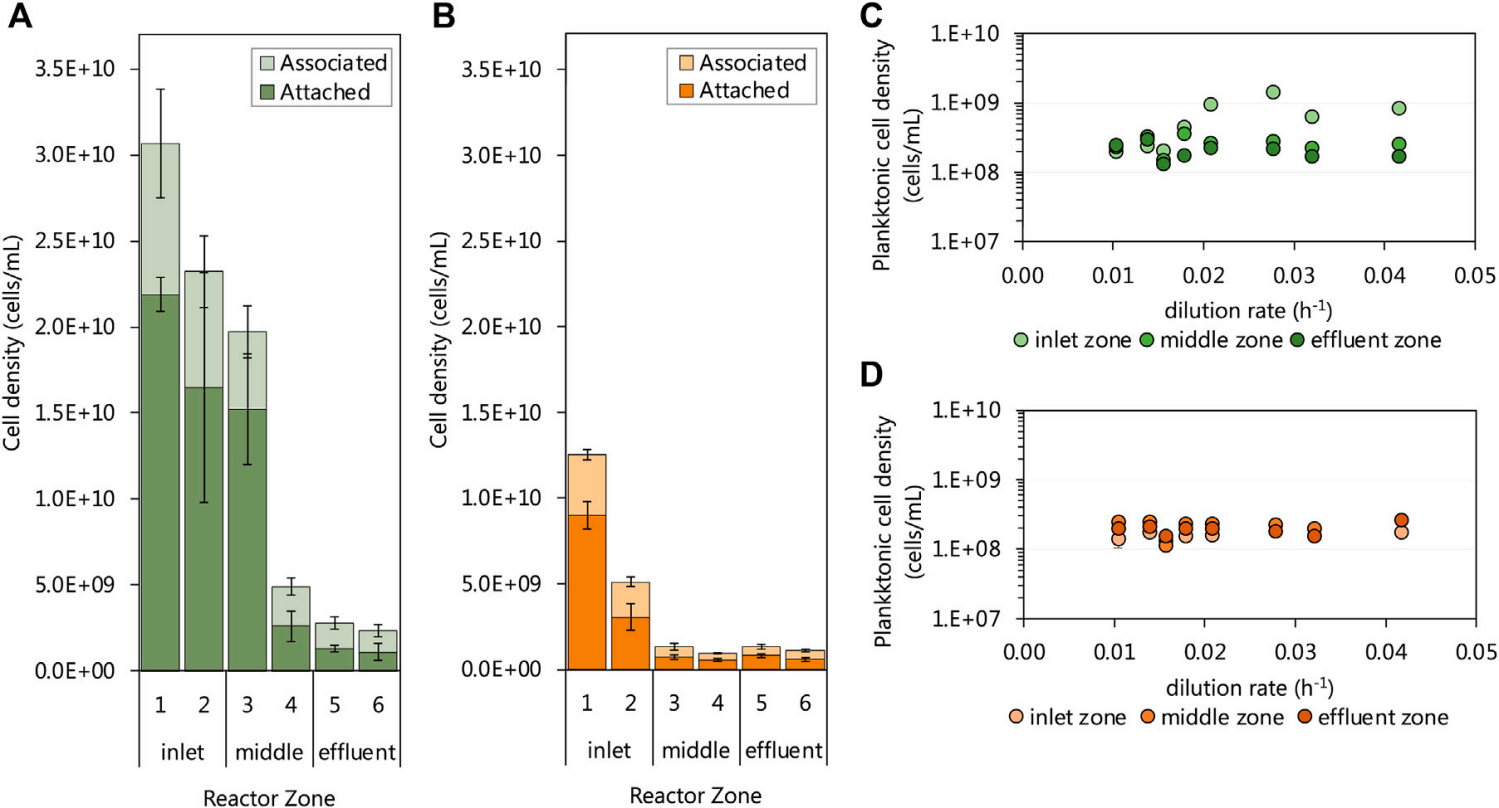
The cell densities of the biofilm-associated and -attached communities throughout the six 0.167 L subzones of the **(A)** lactate and **(B)** acetate UAPBRs at steady-state at a one-day HRT. The steady-state planktonic cell densities from the **(C)** lactate and **(D)** acetate-supplemented UAPBRs inlet-, middle and effluent zones are shown at each tested dilution rate. Error bars represent one standard deviation from the mean with sampling performed in duplicate and each counted in duplicate.

The biomass retained within biofilm communities of each of the six subzones of both UAPBRs was determined at a one-day HRT (0.042 h^−1^ dilution rate). Both UAPBR inlet zones retained high cell densities within the biofilms. Within the lactate UAPBR, the biofilm attached cell density decreased from 2.2 × 10^10^ cells/mL in subzone one to 1.5 × 10^10^ cell/mL within subzone three before decreasing substantially to 2.6 × 10^9^ cells/mL in subzone four (Figure 6A). A similar trend but with lower cell densities was observed in the concentration of biofilm-associated cells across these subzones. However, the ratio of associated to attached cells increased from 0.4:1 in subzone one to 0.9:1 in subzone six.

Within the acetate UAPBR, the cell density of attached and associated biofilm communities was considerable but far lower than supported in the lactate system. The biofilm attached cell density in subzone one of the acetate-UAPBR reached 9.0 × 10^9^ cells/mL and this decreased to 3.1 × 10^9^ and 7.2 × 10^8^ cells/mL in subzone two and three, respectively (Figure 6B). The ratio of associated to attached cells through the subzones of the acetate reactor was similar to that of the lactate system, increasing from 0.4:1 in subzone one to 1.1:1 in subzone six. The extent of the biomass retained in the biofilms in these systems is corresponds with the decreasing number and concentrations of electron donors through each zone.

The density of planktonic cells was assessed at each tested dilution rate and showed little variation with the applied dilution rate, reactor zone or reactor system (Figures 6C,D). One exception to this was the increase in the cell density of inlet zone planktonic community from a 0.021 h^−1^ dilution rate where the cell density increased to 1.46 × 10^9^ cells/mL. This was a result of the level of collected sludge, from falling debris, reaching the height of the inlet zone sampling port. The planktonic concentrations in the acetate and lactate UAPBRs were estimated at an average of 1.89 × 10^8^ and 2.48 × 10^8^ cells/mL, respectively, some orders of magnitude lower than some of the observed cell densities seen in the biofilm communities.

### Microbial Ecology of Planktonic Communities

**FIGURE 7:**
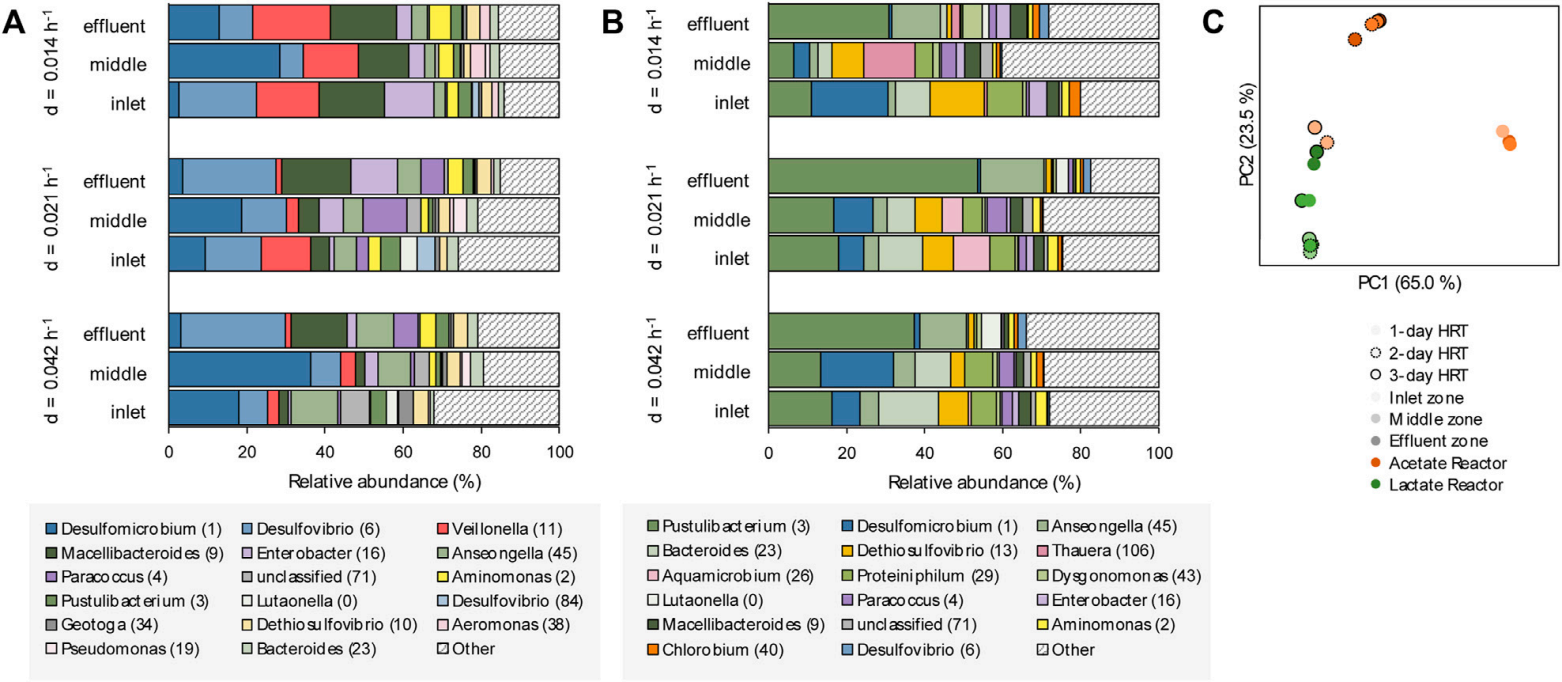
The OTU composition of the planktonic communities of the inlet, middle and effluent zones of the **(A)** lactate- and **(B)** acetate-supplemented UAPBRs isolated at steady-state at a dilution rate of 0.014, 0.021 and 0.042 h^−1^ corresponding to HRT of three-, two- and one-day(s). OTUs are colour shaded by taxonomy: Thermodesulfobacteriota–blue, Pseudomonadota–purple, Bacteroidota–green, Bacillota–red, Synergistota–yellow, Chlorobiota–orange and other phyla–grey. **(C)** Principal component analysis based on weighted Unifrac distances assesses the beta-diversity between these planktonic communities at each HRT.

The planktonic microbial communities of the lactate-supplemented UAPBR, at varied dilution rates, showed little stratification in OTU composition through the height of the reactor (Figure 7A). Some exceptions to this were seen in the acetate-supplemented UAPBBR planktonic communities, where fermentative microorganisms such as the Synergistales *Dethiosulfovibrio* (OTU 13) and the Pseudomonadota *Aquamicrobium* (OTU 26) occurred in the inlet and middle zones at far greater abundances than seen in the effluent zone. No methanogens were detected in any reactor community and is expected to be a result of the use of the methanogenic inhibitor at the time of inoculation.

The composition of both UAPBRs’ planktonic communities also remained fairly stable with increasing dilution rates. However, the abundance of *Veillonella* (OTU 11), classified to a genus implicated in lactate fermentation via the methylmalonyl-CoA pathway (Hilpert and Dimroth, 1991) was elevated at a 0.014 h^−1^ dilution rate, corresponding with greater lactate fermentation observed at this dilution rate.

Principal component analysis of the UniFrac distances between the planktonic communities of these reactors (Figure 7C) found the inlet-zone planktonic communities of the acetate UAPBR to cluster more closely with communities from the lactate UAPBR than others from the acetate UAPBR. This corresponds with the greater representation of the phyla Campylobacterota and Baciliota, and lower representations of Bacteroidota in the inlet zone of the acetate UAPBR, similar to the planktonic lactate communities, compared to the middle and effluent zone communities of the acetate UAPBR.

The planktonic communities of the two UAPBRs demonstrated low SRM diversity with *Desulfovibrio* (OTU 6) and *Desulfomicrobium* (OTU 1) being the only identifiable SRM OTUs found at >1.0% relative abundance. *Desulfovibrio* (OTU 6), however, showed far lower abundances in the acetate-UAPBR and only became abundant within the effluent zone of this reactor.

### Microbial Ecology of UAPBR Communities at a One-Day HRT

**FIGURE 8:**
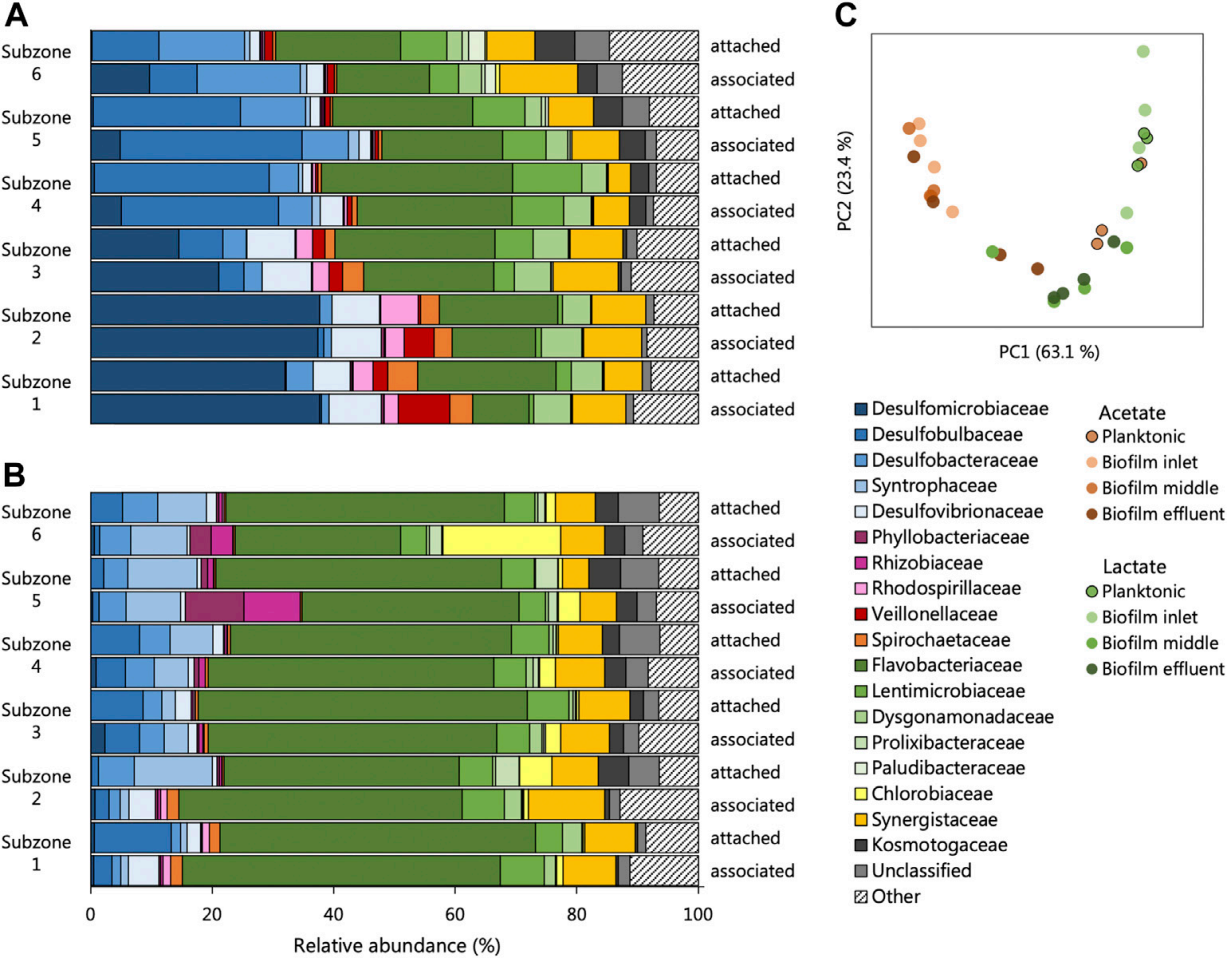
The composition of the attached and associated communities, at the taxonomic family level, of the **(A)** acetate and **(B)** lactate supplemented UAPBRs at a one-day HRT. **(C)** Principal component analysis based on weighted Unifrac distances assesses the beta-diversity between these communities as well as the planktonic communities at a one-day HRT.

Little variation was observed between sequencing repeats (Supplementary Figure S5, Supplementary Table S7) and likely results from the extended time the reactors were allowed to reach true steady states. The alpha diversity of each community, surprisingly, showed few trends between microbial phases, reactor systems and locations within the reactors (Supplementary Table S9). Assessment of the beta-diversity of the UAPBRs’ one-day communities was estimated using weighted Unifrac distances. The PCA of these distances (Figure 8C) exhibited a typical horse-shoe effect indicating the inlet zone biofilm communities were highly dissimilar between reactors (Figure 8C), but similarities were observed between the two effluent biofilm communities where physiological conditions were similar.

The biofilm communities of the lactate reactor showed substantial changes throughout the successive subzones (Figure 8A). Organisms belonging to several Thermodesulfobacteriota families commonly associated with sulfate reduction were found stratified through this reactor: Desulfomicrobiacea and Desulfovibrionaceae were abundant within subzones one to three, Desulfobulbaceae became abundant in subzones four and five, and Desulfobacteraceae become abundant in subzone six. Decreased representation of families not associated with sulfate reduction, through the height of the reactor, included Veillonellaceae, Spirochaetaceae and Rhodospirillaceae, and implicates these microorganisms in competition for lactate, citrate and/or yeast extract. Inversely, there were increases in abundance of Lentimicrobiaceae and the Thermotogae family of Kosmotogaceae throughout the successive subzones which may hint at these being syntrophic acetate oxidisers.

Stratification in the representation of microbial families was far less prevalent in the biofilm communities of the acetate-UAPBR.

**FIGURE 9:**
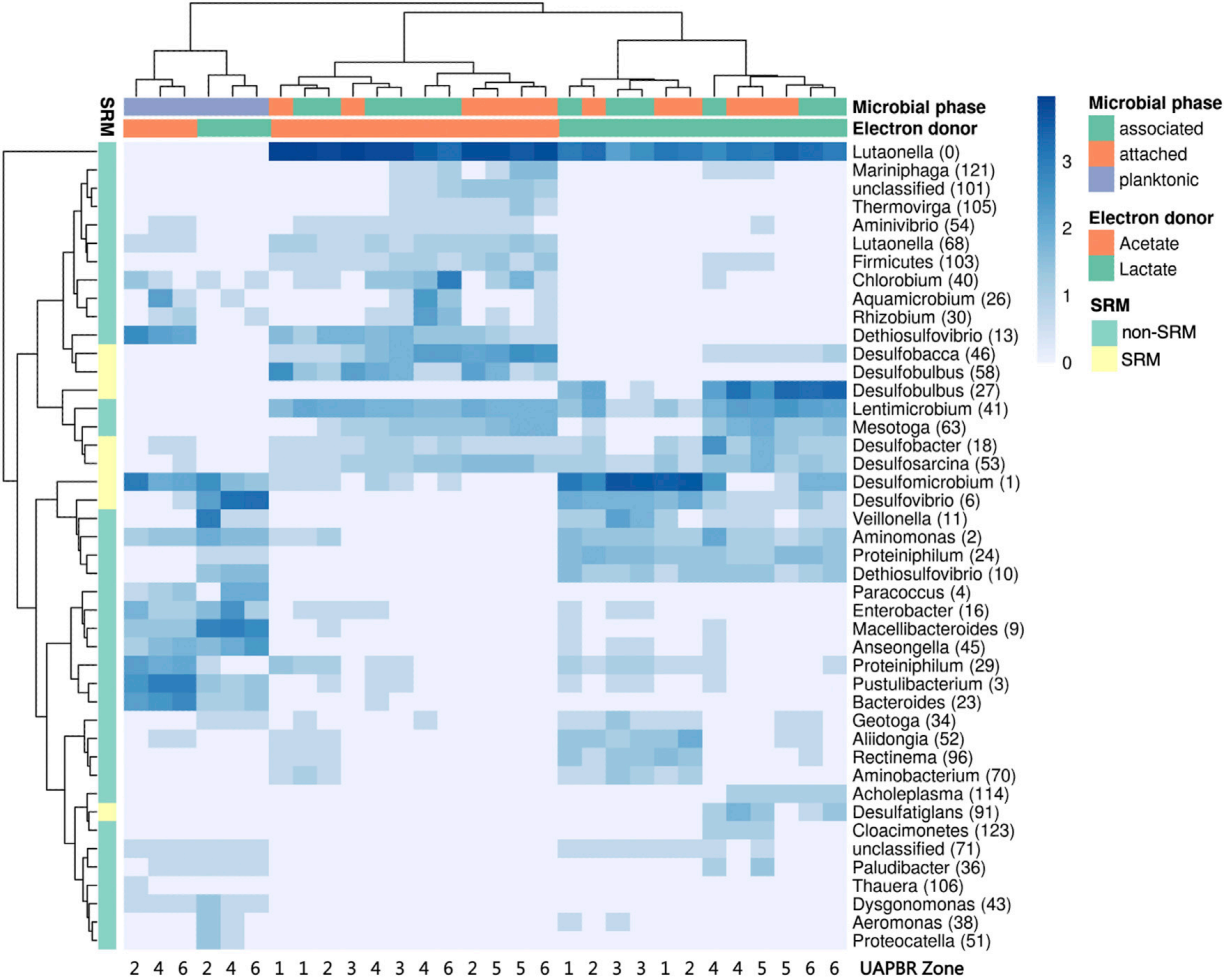
Hierarchical clustered heatmap of log-transformed (ln x+1) relative abundances of predominant OTUs present within the subzones of the acetate- and lactate-supplemented UAPBR planktonic, associated and attached communities at steady-state at a one-day HRT. OTUs putatively identified as SRM based on taxonomy are indicated.

Although some of the communities between the different reactors showed some family-level similarities, the several OTUs which constituted these families and were often themselves restricted to different reactors zones (Figure 9). Hierarchical clustering of the abundances of OTUs across all one-day HRT samples clustered planktonic communities from both reactors clustered together. The planktonic communities had similar abundances of several OTUs including *Desulfomicrobium* (1), *Anseongella* (45), *Enterobacter* (16) and *Aminomonas* (2), among others. Biofilm communities from acetate and lactate UAPBRs each clustered separately. Stratification in the OTU composition of the biofilm communities in each reactor was apparent. *Desulfomicrobium* (1) was the dominant SRM OTU in the lactate UAPBR biofilms in subzones one to three. *Desulfobulbus* (27) then became the predominant SRM in the biofilm communities in subzones four and five and *Desulfobacter* (18) became the predominant SRM in subzone six. The distribution of SRM OTUs throughout the acetate-UAPBR was less stratified with *Desulfobacca* (46), *Desulfobulbus* (58), *Desulfobacter* (18) and *Desulfosarcina* (53) occurring throughout the reactor.

### 16S rRNA Gene Surveys Across the UAPBRs and Four Additional BSR Reactors

In this study we aimed to identify links between reactor performance and the housed microbial communities. We, therefore, sought further evidence to substantiate possible associations between identified OTUs and their favoured growth conditions by using 16s rRNA gene surveys of the UAPBRs and four additional BSR bioreactors that we had simultaneously inoculated and operated under identical conditions.

**FIGURE 10:**
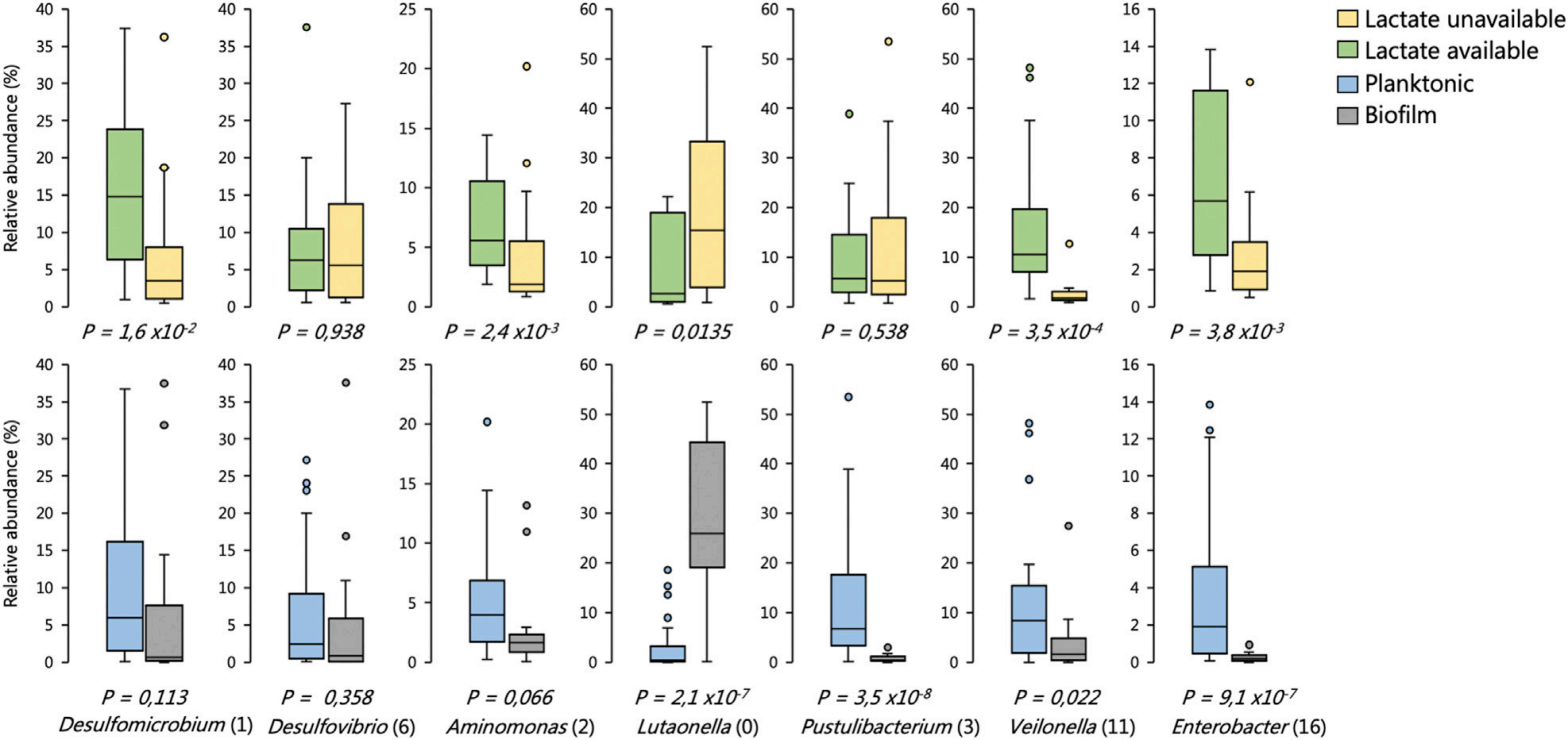
Distribution in the abundance of frequently occurring OTUs across reactor environments of six BSR reactor systems, including environments where lactate was present versus unavailable, and biofilm versus planktonic environments. The box plots show the four interquartile ranges with the maximum and minimum calculated as the interquartile range multiplied by 1.5. Some of these data have been published previously (Hessler et al., 2018; Hessler et al., 2020). Statistical significance was determined by Welch’s test. Sample sizes are shown in Supplementary Table S9.

We found that the SRM *Desulfovibrio* (6) frequently occurred at high abundances across environments regardless of whether lactate was available or not (Figure 10). *Desulfomicrobium* (1), conversely, was significantly enriched in environments were lactate oxidation was occurring (Figure 10). Neither of these OTUs were significantly enriched between planktonic or biofilm communities. The putatively identified competitors of SRM for lactate were *Enterobacter* (16), *Aminomonas* (2) and *Veilonella* (11). Each of these OTUs were enriched in reactor environments where lactate oxidation occurred (Figure 10). *Enterobacter* (16) and *Veilonella* (11) were also found to be enriched in planktonic environments.

The Bacteroidetes OTU *Lutaonella* (0) was one of the few non-SRM OTUs which were significantly enriched in biofilm communities. This OTU was also significantly enriched within zones where lactate was unavailable.

## Discussion

The lactate UAPBR of this study was able to maintain over 90% sulfate conversion at all tested HRT and achieved a maximum VSRR of 40.1 mg.L^−1^. h^−1^, at a one-day HRT. Similar degrees of performance have been observed using other reactor configurations when supplemented with lactate (Elliott et al., 1998; Baskaran, 2005; Baskaran and Nemati, 2006; Kaksonen et al., 2006). In many of these studies, the reactors were able to maintain this level of sulfate conversion at even shorter HRT than we had tested. The noteworthiness of our result stems from this performance being achieved using a far lower concentration of lactate than typically used. The 1.2 g/L lactate was almost entirely consumed within the inlet zone and allowed for a maximum of 0.65 g/L sulfate to be reduced according to incomplete lactate oxidation by SRM (Eq 3). All sulphate which was removed that could not be linked to lactate oxidation was then accounted for based on the observed oxidation of propionate and acetate. The kinetic modelling of the sulfate reduction rates from this reactor found a uniform rate constant could describe the performance throughout the three zones of the reactor. This suggests that the SRM within the middle effluent zones, able to maintain these high VSRRs, were highly adapted to these environments. Biomass quantification of the biofilm cells found these zones to have far lower cell densities than the inlet zone. These results demonstrate that the operating cost of lactate-supplemented BSR reactors can be reduced by limiting the concentration of lactate. This, however, requires the successful cultivation of SRM which are able to perform propionate and acetate oxidation.

Although the overall performance of the acetate UAPBR was comparable to that of the lactate reactor at a 4 day HRT, this reactor was not able to maintain the 97% sulfate conversion at shorter HRT, maintaining a 56% sulfate conversion at a one-day HRT, corresponding with a maximum VSRR of 23.2 mg.L^−1^. h^−1^. This degree of performance has been seen in other studies using acetate as an electron donor (Omil et al., 1997). Yildiz et al. (2019) reported similar sulfate conversions using acetate as the electron donor in a UAPBR but using 2 g/L sulfate, enabling this acetate reactor to achieve VSRR of up to 45.8 mg.L^−1^. h^−1^. Their acetate reactor performed similarly to a second reactor operated with ethanol–another promising result for the use of acetate for BSR.

The inlet zone of the acetate UAPBR of our study, however, did perform similarly to that of the lactate UAPBR for much of the study, despite sulphate reduction coupled to lactate oxidation being a far more favourable than that linked to acetate oxidation (Table 1). This result is partially attributed to the decoupling of the biomass retention and hydraulic retention times within the reactor, as the biofilm communities were by far the highest in cell density within the inlet zone. The performance of the middle and effluent zones were far lower, which was accounted for by the modelled reaction order of 2.9. This had not been anticipated, as several kinetic studies have found acetate-oxidising SRM to have a high affinity for both acetate and sulfate, with characterised saturation constants (*K*_*s*_) in the order of 10^−2^ g/L for both (Ingvorsen et al., 1984; O’Flaherty et al., 1998; Moosa et al., 2002). The reason for the high reaction order has not yet been established, however, these kinetic constants will nevertheless be useful to predict and control for the performance similarly operated reactors.

The planktonic communities of the two UAPBRs showed little stratification, likely a result of plug-flow carrying planktonic cells from the inlet zone throughout the height of the reactor, regardless of cellular activity. The prevalence of the *Veillonela* OTU became reduced following the 3-days HRT, decreasing from 13% relative abundance in the middle zone of the reactor at the 3-days HRT, to just 3 and 1% at the two- and one-day HRTs, respectively. The reduced abundance of this OTU corresponded with the increased proportion of lactate predicted to be utilised via incomplete lactate oxidation by SRM as well as the improved sulphate conversion observed in the inlet zone. We also confirmed that this *Veillonela* OTU was enriched in environments where lactate was present and together these results suggest that this organism is a major competitor of the SRM for lactate.

The majority of the propionate produced in the lactate UAPBR was consumed within it is middle zone at each tested dilution rate and could be putatively linked to the observed sulfate reduction in this zone. An OTU classified as *Desulfobulbus*, a SRM genus commonly associated with propionate oxidation (Hilpert and Dimroth, 1991), was found to be enriched in the biofilms of the middle zone at a 0.042 h^−1^ dilution rate (Figure 9) and is, therefore, likely to be the SRM responsible for this propionate oxidation.

We noted that possible fermentative microorganisms such as the Synergistales *Dethiosulfovibrio* (OTU 13) and the *Aquamicrobium* (OTU 26) occurred in the inlet and middle zones of the acetate UAPBR at far greater abundances than seen in the effluent zone. These OTUs, therefore, are strong candidates for microorganisms which compete for yeast extract components and citrate, the oxidation of which appear to be restricted to the inlet zone of this reactor. Our previous genome-resolved metagenomics study also implicated Spirochaetota in amino acid and citrate fermentation based on encoded metabolism (Hessler et al., 2021). This is consistent with the observed representation of the Spirochaetota in both acetate and lactate UAPBRs inlet zones where the concentrations of these substrates would have been greatest.

The Bacteroidetes OTU *Lutaonella* (0) was one of the few non-SRM OTUs which were found to be significantly enriched in biofilms compared to planktonic communities. This OTU was also significantly enriched within zones where lactate was unavailable and may, therefore, have competed with SRM for acetate. However, due to the abundance of acetate in the reactors and the need to remove residual acetate before discharge, this microorganism was likely beneficial to the overall bioremediation process.

It is often difficult to gain consensus on the role and optimal conditions of particular SRM from 16S rRNA gene surveys of bioreactor systems. This is often due to multiple electron donors being present from the region sampled. The plug-flow which governed our reactors led to the spatially separated environments which made linking SRM to specific conditions in the zone’s in which they were found. Many of the SRM genera identified in the lactate UAPBR are commonly found in other BSR reactor studies using lactate (Kaksonen et al., 2006) and using complex carbon sources (Vasquez et al., 2018). The localisation of the *Desulfobulbus* OTU, specifically in the middle zone strongly suggests this SRM is incompletely oxidising propionate. This association with members of this genus has been made by others before (Hilpert and Dimroth, 1991) and is in agreement with pure culture studies (Stams et al., 1984). A genome of an organism classified as *Desulfobulbus* was recovered from this UAPBR at a four-day-HRT and was confirmed to encode a genes necessary for propionate utilisation. *Desulfosarcina* (53), and *Desulfatiglans* (91) were identified SRM which became abundant in the final three subzones of the lactate reactor and are expected to be performing the acetate-oxidation observed in these zones.

The distribution of SRM OTUs throughout the acetate-UAPBR was less stratified with *Desulfobacca* (46), *Desulfobulbus* (58), *Desulfobacter* (18) and *Desulfosarcina* 53) occurring throughout the reactor. The acetate oxidation pathways and pure-culture growth kinetics using acetate of representatives from these genera have been assessed in several studies (Ingvorsen et al., 1984; Schauder et al., 1986; O’Flaherty et al., 1998; Oude Elferink et al., 1999). Their dominance in the acetate UAPBR indicates their capacity to effectively compete for acetate in these mixed microbial bioreactor environments.

It is possible that some of the identified SRM could be consuming citrate or yeast extract. In particular it was noted that *Desulfovibrio* (6), was abundant in several environments irrespective of whether lactate was available, and may therefore be relying on these electron donors. However, the strong agreement between our electron donor predictions based on the sulfate and VFA concentrations suggests that if sulfate reduction is linked to oxidation of these electron donors is likely to be minimal. This is also substantiated by the limited short- and long-term effect on performance of the reduction in yeast extract from 1.0 to 0.4 g/L before the start of the HRT study (Hessler et al., 2021). It is also possible that *Desulfovibrio* (6) may effectively compete for both lactate and acetate in mixed communities. The oxidation of both lactate and acetate by *Desulfovibrio* species has been reported. Liu et al. (2018) amended soils rice paddy sediments with ^13^C acetate, and observed that *Desulfovibrio* was one genera which become stimulated and incorporated ^13^C in its nucleic acids. Sorokin (1966) first noted that acetate was assimilated into *Desulfovibrio vulgaris* biomass when growing on hydrogen. This is one form of metabolism which we suspect may be occurring in our bioreactors which we hadn’t initially considered. The previous genome-resolved metagenomic analysis of the acetate-oxidising bioreactor communities too suggest bio-hydrogen, produced through fermentation of lactate, citrate and yeast extract, could be promoting the growth of SRM which all encoded numerous hydrogenases (Hessler et al., 2021). Further investigation is required to validate this hypothesis.

A common feature to both UAPBRs was the greater SRM diversity in the biofilm over planktonic communities. This indicates that these biofilm-SRM are not able to effectively compete with planktonic microorganisms which leads to their washout. This is an important observation as increased microbial diversity has been associated with resilience to system perturbation in other bioprocesses (Feng et al., 2017). We, therefore, recommend the limiting of planktonic communities and the promotion of biofilm communities in BSR reactors to favour lactate-oxidising SRM over fermenters, enhance SRM diversity and promote the growth and volumetric activity of slow-growing acetate-oxidising microorganisms.

## Data Availability

The datasets presented in this study can be found in online repositories. The names of the repository/repositories and accession number(s) can be found in the article/Supplementary Material.
