# Supplementary material for: Integrated Kinetic Modelling and Microbial Profiling Provide Insights Into Biological Sulfate-Reducing Reactor Design and Operation
Source: Front Bioeng Biotechnol. 2022 Jun 29;10:897094. doi: 10.3389/fbioe.2022.897094 (PMC9277144; doi:10.3389/fbioe.2022.897094)
Supplement: Supplementary file 1 [file DataSheet1.PDF]

Supplementary methods: Derivation of kinetic equations

The sulfate reducing performance of each UAPBR was modelled as ideal plug flow reactor according to Eq (1), where *X* is the sulfate conversion, *V* is the volume of the reactor or zone, *rA* is the sulfate reaction rate, *F* is the flow rate, C_0 is the concentration of sulfate entering- and C_A the concentration of sulfate leaving the reactor or zone. A general rate Eq (2) was substituted into Eq (1) to produce an expression describing the rate of an nth order irreversible reaction, with rate constant *k*, in an ideal plug-flow reactor, where C_A is the concentration of substrate leaving the reactor or zone (Eq 3). Eq (1) was resubstituted into Eq (3) to eliminate the dX term (Eq 4). Rearranging Eq (9), performing integration and solving for C_A produces Eq (5). This could be substituted into a formula describing the volumetric reaction rate over a volume of reactor (Eq 6). Finally, this produced equation describing the rate of an irreversible n^{th} order reaction with a rate constant *k,* over a volume (*V*) of an ideal plug flow reactor (Eq 7) which could vary depending on the sulfate concentration (C_0) entering the zone and flow rate (*F*) applied. However, Eq (7) cannot be used to describe a first-order reaction as an n term equal to 1 causes the exponent of the term shown in Eq (4) within Eq (7) to become undefined. Therefore, the derivation described above was performed as second time using the same rate equation (Eq 7) but making n equal to one. This gave a similar equation to Eq (8) but which could only be used to describe first-order reactions.

$$\frac{dX}{dV} = \frac{-rA}{F.C_0} \qquad \text{Eq (1)}$$

$$r_A = -k.C_A{}^n \qquad \text{Eq (2)}$$

$$\frac{dX}{dV} = \frac{-k.C_A{}^n}{F.C_0} \qquad \text{Eq (3)}$$

$$\frac{-\frac{1}{C_0}\,dC_A}{dV} = \frac{-k.C_A{}^n}{F.C_0} \qquad \text{Eq (4)}$$

$$C_A = \left(C_0^{(-n+1)} + (n-1)\cdot k \cdot \frac{V}{F}\right)^{\frac{1}{(-n+1)}} \qquad \text{Eq (5)}$$

$$rA = -\frac{F}{V}(C_A - C_0) \qquad \text{Eq (6)}$$

$$rA = \frac{V}{F}\left(\left(C_0^{(-n+1)} + (n-1)\cdot k \cdot \frac{V}{F}\right)^{\frac{1}{(-n+1)}} - C_0\right) \qquad \text{where } n \neq 1 \qquad \text{Eq (7)}$$

$$rA = -\frac{F}{V}\left(\frac{C_0}{e^{\frac{V.k}{F}}} - C_0\right) \qquad \text{Eq (8)}$$

Experimental data collected from each UAPBR was used to solve for the rate constant and reaction order of the overall sulfate reduction reaction observed in each reactor over the HRT study. The applied flow rate (*F*); the volume of reactor zone or composite zone (*V*; eg inlet and middle zone of 0.66 L); the

concentration of sulfate entering the zone(s) (C_0); and the observed reaction rate calculated using Eq (1) associated with each data point were substituted into Eq (7) and Eq (8).
